# Eight energy and material flow characteristics of urban ecosystems

**DOI:** 10.1007/s13280-016-0785-6

**Published:** 2016-05-10

**Authors:** Xuemei Bai

**Affiliations:** Fenner School of Environment and Society, Australian National University, Canberra, ACT 2601 Australia

**Keywords:** Urban metabolism, Urban ecosystem, Energy and material flows, Cross pollination, Integrated urban theory

## Abstract

Recent decades have seen an expanding literature exploring urban energy and material flows, loosely branded as urban metabolism analysis. However, this has occurred largely in parallel to the mainstream studies of cities as ecosystems. This paper aims to conceptually bridge these two distinctive fields of research, by (a) identifying the common aspects between them; (b) identifying key characteristics of urban ecosystems that can be derived from energy and material flow analysis, namely energy and material budget and pathways; flow intensity; energy and material efficiency; rate of resource depletion, accumulation and transformation; self-sufficiency or external dependency; intra-system heterogeneity; intersystem and temporal variation; and regulating mechanism and governing capacity. I argue that significant ecological insight can be, or has the potential to be, drawn from the rich and rapidly growing empirical findings of urban metabolism studies to understand the behaviour of cities as human-dominated, complex systems. A closer intellectual linkage and cross pollination between urban metabolism and urban ecosystem studies will advance our scientific understanding and better inform urban policy and management practices.

## Introduction

The concept of urban metabolism has been widely used to study energy and material flows into and out of cities, with a rapidly growing body of literature over the last 10 years (Decker et al. 2000; Warren-Rhodes and Koenig 2001; Kennedy et al. 2007; Zhang and Hu 2011). It has started as a metaphor of likening cities to a living organism, and while there are precursors of such thinking (Fischer-Kowalski and Hüttler 1998), modern use of the concept was pioneered by Abel Wolman in his study of an imaginary city of 1 million people, looking at total resource inputs into the city, and waste output from the city (Wolman 1965). The extended urban metabolism concept encompasses four elements: the total input (e.g., energy, material, money, information), distribution of the input within city to drive urban functions, the total output (e.g., products, emissions, knowledge), and the regulating function that shapes such flows and distributions (Bai and Schandl 2010). Numerous empirical studies were conducted in cities worldwide (Newcombe et al. 1978; Boyden et al. 1981; Baccini and Brunner 1991; Hendriks et al. 2000; Warren-Rhodes and Koenig 2001; Tarr 2002; Huang et al. 2006; Browne et al. 2011; Kennedy et al. 2015). Adopting a comprehensive urban metabolism accounting approach, or focusing on individual substances of interest, and ranging from household to neighbourhood to city level, these studies revealed the large and increasing global impacts of cities (Bai 2007).

The concept has been found intriguing and useful (Decker et al. 2000), and has indeed inspired numerous empirical studies. These studies served as important means to inform policy and management by presenting the stocks and flows of resources and environmental impacts in a quantified, easy to understand manner. However, the concept has also been subject to criticism and debate. Firstly, research has tended to focus predominantly on quantifying various flows in and out of cities, without critical analysis of the concept (Lifset 2004; Swyngedouw 2006), or conscious effort to build upon and extend beyond empiricism—little attention has been paid to understanding how such approach and accumulated empirical evidences can contribute to the ecological insights of understanding cities as complex social-ecological systems, or how to enable and extend such contributions. This raised the question of whether urban metabolism studies can offer any insight beyond a series of numbers from accounting exercises, or contribute to needed theoretical development in urban research. Secondly, it has become a widely accepted notion that more than an organism, cities are human-dominant, coupled, complex ecosystems (Grimm et al. 2000; Alberti et al. 2003; Cadenasso et al. 2006; Liu et al. 2007; Grimm et al. 2008), as discussed in detail in the following section. This led to the questioning of the appropriateness of the metaphor and analyses driven by it (Golubiewski 2012).

However, recent urban energy and material flow studies have extended far beyond the original metaphor of cities as organism, and started to reveal important characteristics of urban system features and interactions. While both argue for the need of more integrated approaches in urban research, the urban material and energy flow research community, and the urban ecosystem research community are not sufficiently linked to achieve such integration. The potential linkages between these two communities are recognized before (Bai, 2007; Bai and Schandl, 2010), but previous studies have stopped at pointing out the issue and comparing the approaches. More recent debates (Golubiewski 2012; Kennedy 2012) indicate there are still unresolved tensions and gaps between these two approaches.

There are at least four reasons to move beyond and look for common ground: (a) cities are unique ecosystems, in that they are human-dominated with strong regulating and governing mechanisms, which cannot be fully explained by existing ecosystem concepts, theories, and approaches developed from the study of natural ecosystems; (b) ecological processes in urban systems are strongly influenced by anthropogenic resource flows, which are the primary focus of urban metabolism studies; (c) as discussed later in this paper there are many commonalities between urban ecosystem research and metabolism research than the branding might suggest; and (d) the rich empirical evidence and some recent trends in urban metabolism studies might have the potential to reveal key characteristics of urban ecosystems.

To mature and resolve this debate constructively, the following questions need to be asked. Are these two approaches mutually exclusive and irreconcilable? What are the similarities and differences between urban metabolism studies and urban ecosystem studies? Can studies undertaken under the banner of urban metabolism contribute to understanding cities as ecosystems? Here I argue that there are more commonalities between urban ecosystem and urban metabolism approaches than the difference in conceptualization may suggest. I then present eight key energy and material flow characteristics of an urban system, our state-of-the-art understanding about them, and their ecological and practical significance. I stress that a closer intellectual linkage and cross pollination between the two can not only contribute to the much needed theoretical development on cities as human dominant system, but also better inform urban policy and management practices, e.g., to avoid unintended consequences.

## Cities as unique ecosystem

**Fig. 1:**
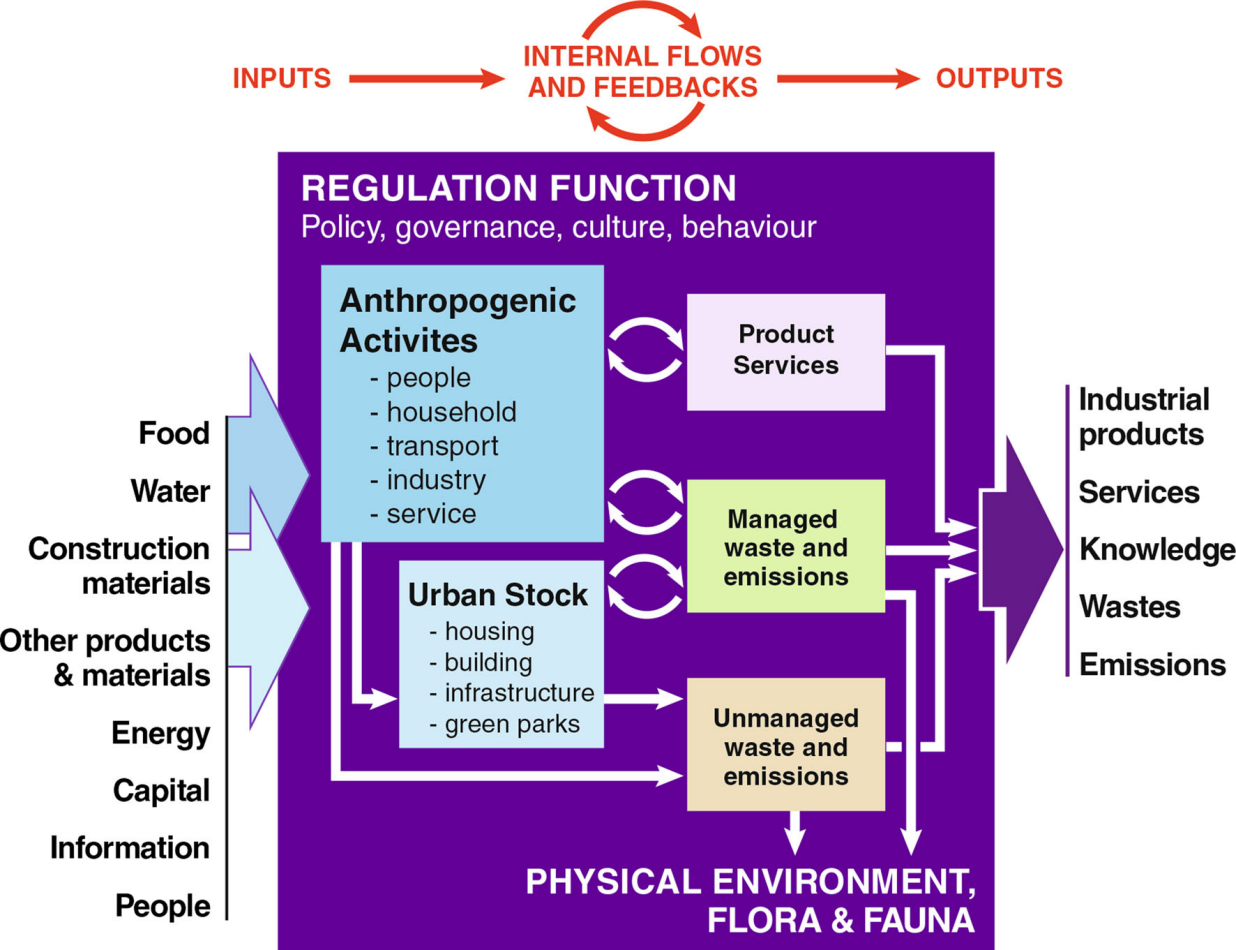
Conceptual diagram of urban metabolism. Part of the resources flow into cities become urban stock, others enable and drive various anthropogenic functions, and eventually produce intended or unintended outputs that either stay within the system boundary or exported beyond the boundary, with various impacts on the physical environment, flora and fauna and associated ecological processes. Urban metabolism is shaped and regulated by factors such as urban policy and governance, culture, and individual behaviours

Cities are ecosystems, but they are very different from natural ecosystems. An urban ecosystem is human-dominated, and is governed by complex interactions among components as well as a unique regulating and governing mechanism that shape social and ecological processes (Faeth et al. 2005; Andersson 2006; Kaye et al. 2006; Bai 2007). The resources that flow into cities shape and alter the structure of urban ecosystem, enable, and drive urban functions with influence on natural ecological processes of cities, and eventually produce intended or unintended outputs that either stay within the system boundary or exported beyond the boundary. Figure 1 shows the conceptualization of urban ecosystem from material and energy flow perspectives. The input part of the urban metabolism includes various tangible materials such as food, water, construction and other materials, products, energy, as well as inflow of energy, capital, information, and people. Such input supports societal activities and drives urban functions within a city; forms urban stocks such as housing, building, infrastructure, and green parks; and produces products and services, as well as managed and unmanaged waste and emissions. The output part consists of industrial products, services, knowledge, and various wastes and emissions. The magnitude, distribution, and internal interactions and feedbacks are regulated by policy, governance, culture, and individual and collective behaviour of the urban system.

The human dominant feature of urban system means the concepts, theories, and approaches developed for, and knowledge obtained from, natural ecosystems are unlikely to be sufficient to explain an urban ecosystem. For example, biogeochemical cycles of nutrient such as C, N, P, or the flow of energy through food system, are a key focus of ecosystem ecology, but they only comprise a small part of the large variety and magnitude of materials or energy flows in an urban system. In addition, while natural ecosystems may not have “a set points of control” (a number of quantities the organism tries to keep at a particular value) like organisms (Odum et al. 2005), cities as human dominant systems do have stronger regulating and governing functions and mechanisms, such as the existence of city level government, which is embedded within the country’s government system and linked across various actors and agencies within and beyond the city. This regulating and governing mechanism plays a critical role in urban ecosystems, through making policy, planning, and management decisions that influence both anthropogenic and ecological processes within and beyond the city. The ultimate goal of understanding urban ecosystems is to use such understanding to guiding sustainable development of cities, and such application often need to be realized through the regulating and governing functions of cities.

While the current scope of the urban ecosystem studies includes both anthropogenic and anthropogenically dominated ecological processes within cities (Pickett et al. 2011; Pincetl 2012), studies have focused more on anthropogenically affected ecological processes, rather than anthropogenic processes. There is a need to focus more on human endeavour itself in urban ecosystem studies. Integrating humans into the system is widely recognized as important at conceptual level, but developing effective and integrative theories and approaches for urban system study remains a challenge (Collins et al. 2000; Alberti et al. 2003; Pickett et al. 2004; Coelho and Ruth 2006). We need to continue searching for the key characteristics of cities as ecosystems, examine how effective different perspectives in revealing them, and adopt and develop different concepts, theories and methods accordingly. The uniqueness of urban system may well require the coexistence of different perspectives to explain different characteristics of cities.

## Urban ecosystem and urban metabolism: Irreconcilable approaches?

**Fig. 2:**
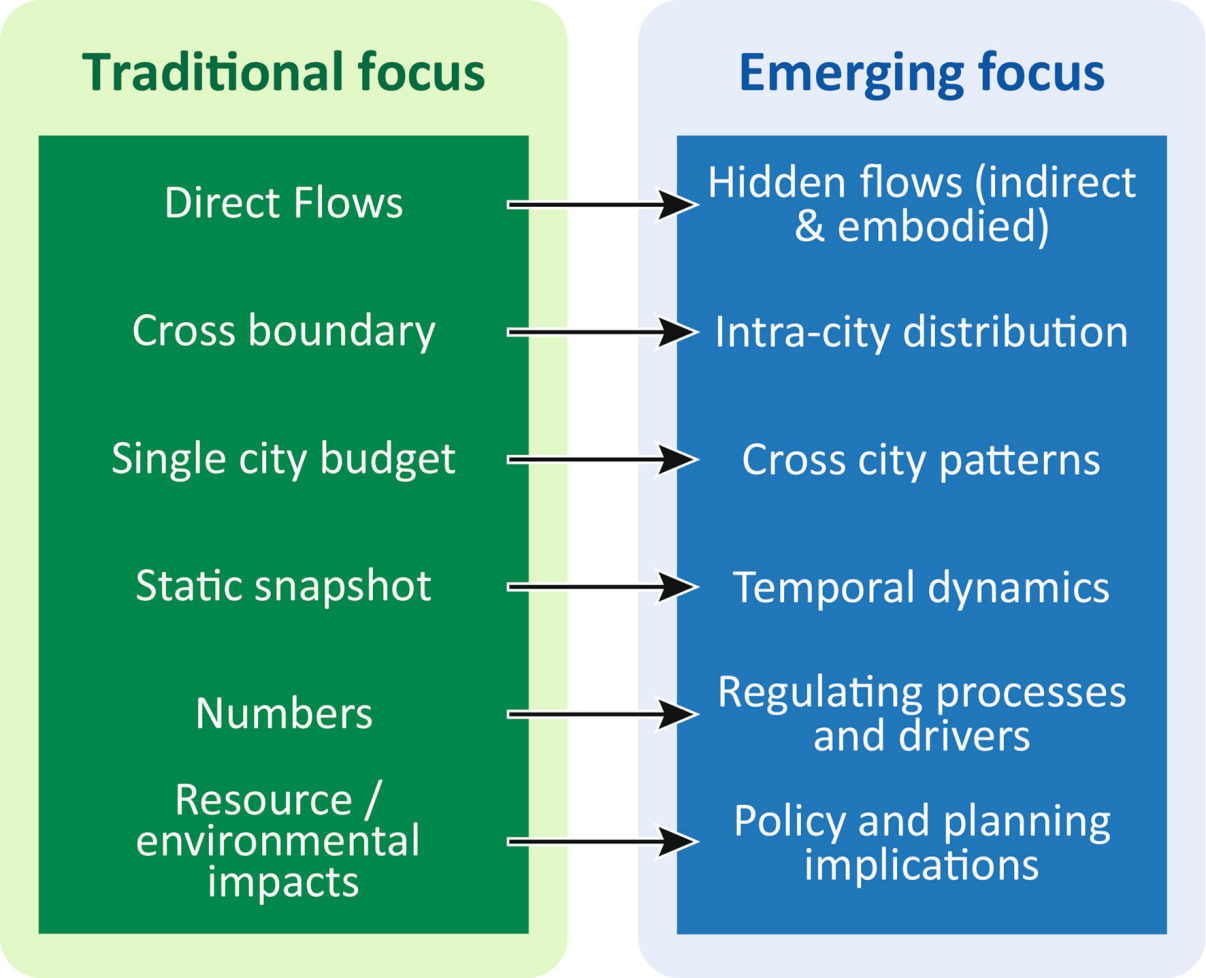
Traditional and emerging focus in urban metabolism studies. Emerging trend in recent decade shows a shift away from static snapshots of cross boundary direct material and energy flows, towards more comprehensive and dynamic accounting, as well as the drivers and policy implications

While with very different conceptual starting points the two bodies of literatures on urban metabolism and urban ecosystem studies share significant common attributes, some early-stage urban metabolism studies were undertaken as urban ecosystem studies under the UNESCO Man and Biosphere Program in the 1970s, which formed early foundations of urban ecosystem approach, and the term “urban ecosystem” is frequently used in urban metabolism studies (Decker et al. 2000; Bai and Schandl 2010; Zhang and Hu 2011). The past decade has witnessed a rapid growth and a significant shift of focus in urban metabolism research (Fig. 2), which can be summarized as follows:

- *From direct flows to hidden flows* In addition to the traditional interests in the direct flows of materials and energy, recent studies pay more attention to hidden flows of energy and materials through cities, which are the flows that are included in the goods and services cities consume or produce. This reflects the reality that modern cities might be increasingly capable of reducing direct energy and material flows, but at the same time they are increasingly dependent on energy and material intensive processes elsewhere that produce the goods and services they consume. Many studies show that direct flows consist of rather small part of total urban energy and material footprints (Schulz 2010; Lin et al. 2013).
- *From cross boundary to intra*-*city distribution* Earlier metabolism studies treated cities almost as a ‘black box’, and only considered the flows in and out of the boundary. Recently, more attention is paid to intra-city distribution of the flows, in terms of sector, socioeconomic variables, or spatial pattern. This looks inside the black box, and attempts to explore the structural determinants of the urban system in relation to metabolic flows.
- *From single city budget to cross city patterns* Most earlier urban metabolism studies are single city-based comprehensive budget accounting. With the accumulation of single city analysis, and the increasing availability of city level data, more studies start to explore cross city patterns. This enables cross city comparison and bench marking of resource and environmental performance, as well as exploring the functional differences of cities.
- *From static snapshot to temporal dynamics* Traditional urban metabolism analysis provides a static snapshot of resource input and environmental output of cities, but recent literature increasingly focus on changes over time. This enables better links between a city’s resource and environmental impacts to its development or evolutionary processes, and to the evaluation of the effectiveness of policy and management decisions.
- *From numbers to regulating processes and drivers* Some recent literature attempts to link the numbers obtained from metabolism analysis to regulating processes and drivers such as consumption behaviour or policy measures, as illustrated in the following section, although much more effort is needed to establish such linkages.
- *From resource and environmental impacts to policy and planning implications* Urban metabolism analysis quantifies the resource and environmental impacts of cities. Due to its often heavily technical and quantitative approach, findings from urban metabolism research are not often used in urban ecological studies, nor effectively used to inform urban planning and policy making practices, although there is a growing aspiration to do so. Simply adopting more innovative ways of presenting the results in a user friendly way might assist such applications.

At the same time, urban ecosystem studies has also shifted from primarily focusing on “ecology in cities”, concerned with flora and fauna of cities, towards looking at cities as ecosystems and integrating humans as part of the ecosystem cities as ecosystem, as exemplified by the concepts of “ecology of cities” or “urban social-ecological systems”. As a result, there is a converging trend between urban metabolism approaches and urban ecosystem approaches.

**Fig. 3:**
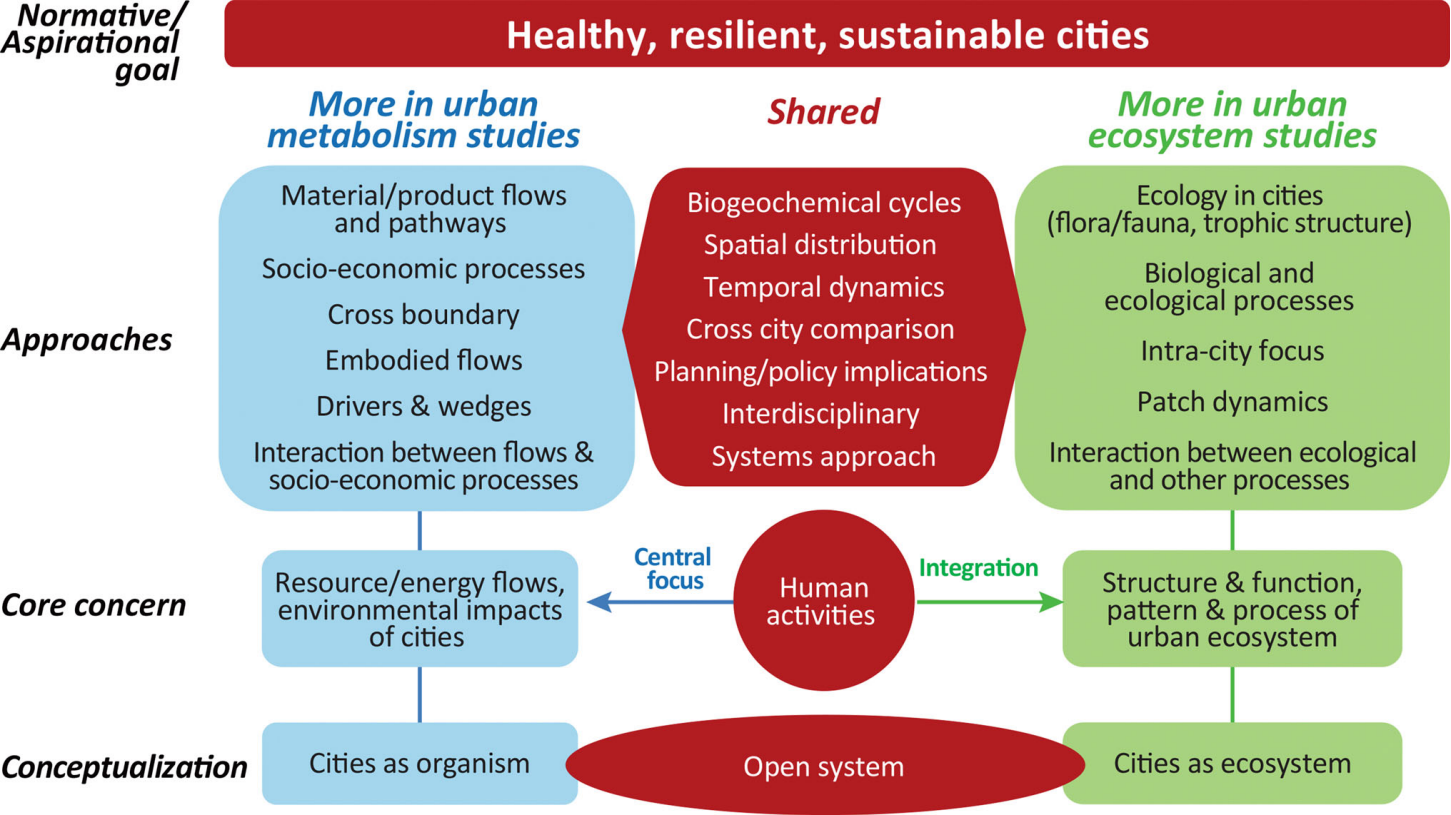
Unique and shared features of urban material and energy flow analysis and urban ecosystem studies. With very different conceptual starting points the actual body of literature shares significant common attributes. The items included under each column are based on relative comparisons, recognizing there is a continuum in the degree of emphasis. The emphasis is on the shared components and a common goal

Figure 3 shows shared and unique aspects of urban metabolism and urban ecosystem studies. The items included under each column are based on relative comparisons and not necessarily exclusive, and it is important to recognize there is a continuum in the degree of emphasis.

At the conceptual level, urban metabolism adopts the metaphor of “cities as organism”, while most urban ecologists adopts “cities as ecosystem” perspective. However, more than anything else, the essence of the organism metaphor lies in emphasizing the relations between cities and their hinterland as inseparable, just as an organism within their environment. This *open system* feature of cities is shared in both urban metabolism and urban ecosystem perspective.

In terms of the core concern, the urban metabolism studies are predominantly motivated by the external impact of the system—the resource and environmental impact of cities—and therefore more emphasis is on the interaction between cities and their hinterlands. On the other hand, urban ecosystem studies are motivated to understand the structure, function, patterns, and process of urban ecosystem, and hence more emphasis is on the system itself. Both share keen interest in the human component; human activities are at the centre of concern for urban metabolism analysis, and with the shift from “ecology in cities” to “ecology of cities”, and the focus on urban social-ecological systems studies, integrating humans into the system has become increasingly important in urban ecosystem studies (Grove and Burch, Jr. 1997; Grimm et al. 2000; Elmqvist et al. 2004).

More overlaps can be found in the approaches. Both literatures show strong interest in biogeochemical cycles, spatial patterns and temporal dynamics, cross city comparison, adopt interdisciplinary and systems approaches, and both aim to draw implications for urban planning and policy. Meanwhile, urban metabolism studies are more concerned about material and energy budget and pathways focusing on anthropogenic processes, cross boundary interactions including accounting for the embodied flows beyond city boundaries, and the drivers and particular impacts of processes on the various aspects of the flows. Unique aspects of urban ecosystem studies as contrast to urban metabolism include traditional ecology in cities such as urban flora and fauna, trophic structure, and biological and ecological processes and their interactions with other social economic processes (Faeth et al. 2005), which tend to be more intra-city focused. Urban ecosystem studies have unique approaches such as patch dynamics to understand the interactions among spatially distributed ecological components (Wu 2008).

Last but not least, both bodies of studies share the normative aspirational goal, i.e., contributing towards healthy, resilient, and sustainable cities.

## Eight material and energy characteristics of cities

Recent urban metabolism studies have extended far beyond the original approach to quantify the flow budget, and started to reveal important characteristics of urban system features and interactions. Here I present some important characteristics of an urban ecosystem that can be derived from urban metabolism studies, which is categorized into eight aspects (see Table 1). Some of these characteristics are by no means exclusive to urban metabolism studies, but these studies can complement the findings from urban ecosystem studies and strengthen the empirical basis. For each of the eight features, key research questions, normative goals in light of urban sustainability, and the level of existing empirical evidences are also presented.

**Table 1:** Eight material and energy characteristics of urban ecosystems

Characteristics of urban ecosystem	Key questions	Sustainability goals	Level of empirical evidences
Material and energy budget and pathway	What type, how much total flows, and via what pathways? What are the global impacts of such flows?	Lower total budget	•••
Material and energy intensity	How intensive are the flows, measured as flows per capita or per area?	Lower intensity	••
Material and energy efficiency	How much social/economic services can per unit of resource consumption or waste generation support?	Higher efficiency	••
Rate of accumulation and retention	How much of the input remains in urban system? How much is exported? How long does the inflow material stay within the system?	NA	•
Self-sufficiency (external dependency)	To what extent the urban system’s resource needs are met internally or externally?	Higher self-sufficiency	•
Intra-system heterogeneity	How the above indicators distribute within the system? How and/or spatial structure of urban system determine/affect such heterogeneity?	Lower social economic-related variation	••
Temporal and intersystem and variation	How the above indicators change across cities and over time? How different cities bench mark against each other?	Improving trend	••
Regulating capacity	What are the regulating mechanisms of the flows (e.g., policy, management, interactions among system components), and what are their capacity and limitations?	Effective use of the potential	•

The ecological significance, key findings, and where possible policy implications of each of the eight characteristics are discussed below.

### Material and energy budget and pathway

Understanding biogeochemical budgets of ecosystems, in particular nutrients such as carbon, nitrogen, and phosphorus, has been one of the crucial elements of urban ecology (Pickett et al. 2011). However, there is a need to expand such flux analyses from biogeochemical elements to materials, as in an urban system, there are much more diverse flows, and the non-nutrient material flows far exceed the traditional nutrient element flows in volume (Warren-Rhodes and Koenig 2001; Niza et al. 2009). In an urban ecosystem, anthropogenic flows far exceed those mobilized by natural processes (Brunner 2007; Zhang et al. 2012). The human-subsidized resource and energy flows in cities have significant ecological consequences, such as reduced or increased number of wildlife species (DeStefano and DeGraaf 2003). The metabolic budget can be used to assess the total ecological footprints of cities (Moore et al. 2013), which is identified as one of the key elements of urban ecosystem studies (Pickett et al. 2001). There are large throughputs of material in an urban system, which are not necessarily produced or consumed in the city (Vause et al. 2013), which is another unique characteristics of urban system. The total budget and pathways of material and energy flows reveal the magnitude of impacts and other important characteristics of urban system, such as the functional role of the city, development stages (i.e., mature or growing city), level of infrastructure and development, income, and other socioeconomic characteristics of the city (Hu et al. 2010a, b; Browne et al. 2011; Li et al. 2012; Miller et al. 2013).

### Material and energy intensity

Energy and material flow intensities, which is often measured by per area or per capita in the case of cities, are important indicators for an ecosystem. Urban areas are much more energy intensive than natural ecosystems. Globally, even if all urban areas are covered with solar panels, they cover only about 2 % of energy requirements of cities (Grubler et al. 2012). Some biogeochemical fluxes, even though only representing part of total material flows in cities, are much more intensive than natural or even heavily managed and subsidized agricultural systems. A study in Xiamen City, China shows the inflow intensity (load of imported P per unit urban area) of urban dietary P is two to three times higher than that of chemical P fertilizer application on agricultural land, with a high-accumulation ratio (Li et al. 2012). Anthropogenic carbon flux in cities is about 10–100 times larger than natural sequestration capacity through net primary productivity (Pataki et al. 2011). In addition to the flow intensity, the behaviour of material and embodied energy stock per unit area in urban system, including whether there is a saturation level, can be important to explore in order to understand and forecast future potential for recycle and inflow needs (see for example (Tanikawa and Hashimoto 2009)), but very little empirical evidence exists.

### Material and energy efficiency

Urban energy and material flow efficiency can be defined as how much social/economic services per unit of resource consumption or waste generation can support. It shows how efficient the urban system is in supporting its function, and is an important system performance indicator. Although higher efficiency sometimes can be detrimental to other system performance such as resilience, the high material and energy intensity of urban system is directly linked to significant environmental impacts, therefore enhancing efficiency can often become an important policy goal. Within the urban metabolism literature it is measured in two different ways: the amount of economic output or social services generated by per unit resource consumption or per unit emission (Zhang and Yang 2007; Vause et al. 2013); or the ratio of waste disposal as a function of product consumption (Browne et al. 2009). There are large disparities in flow efficiency over time and across cities. Empirical evidence shows an improvement in metabolic efficiency by up to 3.7 times by the first measure in Shenyang during 1998–2004 (Zhang and Yang 2007), and a 31 % improvement by the second measure in Irish city region during 1996–2002 (Browne et al. 2009). While technology, infrastructure, transportation systems, density, and consumer behaviour of cities are some of the better known factors that affect energy and material efficiency of a city (Weisz and Steinberger 2010), evidence shows that urban green infrastructure can reduce energy demand in cities (Bolund and Hunhammar 1999). The intersection between urban natural ecosystems and the energy and material efficiency of urban functions deserves more attention from both urban metabolism and urban ecosystem research.

### Speed of flow and rate depletion/accumulation

Cities are increasingly becoming “reservoirs” for both resources and pollution, (Warren-Rhodes and Koenig 2001; Brunner and Rechberger 2002; Kapur and Graedel 2006). How long does it take for a certain material to flow through urban ecosystem How much of the flows are retained and accumulated within the system? How much of it is degraded through the process? These are important questions to answer, in order to understand future waste flows and exploring the potential to consider “cities as future mines”. There is a large disparity in total accumulation, retention rate, and flow speed for different materials and across different cities or over different stages of urban development (Niza et al. 2009; Tanikawa and Hashimoto 2009; Hu et al. 2010a, b). High level of concentration of materials can change the morphology and spatial structure of cities, and material concentration can occur underground at roughly the same magnitude as aboveground (Tanikawa and Hashimoto 2009). High levels of retention/accumulation of materials and energy, such as the accumulation of nutrient elements in urban soil and water bodies (Warren-Rhodes and Koenig 2001; Li et al. 2012), may alter urban ecosystem in various ways, but there is little empirical evidence describing these linkages beyond those traditionally identified as pollutants.

### Self-sufficiency versus external dependency

Cities are open systems with high dependency on their hinterlands, which range from local, regional to global. The level of self-sufficiency or external dependency can be an important indicator to understand the resilience of an urban system. The external dependency of cities can be reduced and self-sufficiency be enhanced by effectively mobilizing the resources stocked or flowing through the city. Recent urban metabolism literature shows a large potential: for some resources, over 50 % and up to a 100 % of self-sufficiency is possible by quality differentiation of resources and innovative collaboration between the public and private sectors within the city (Baccini 1997; Beatley 2007; Agudelo-Vera et al. 2012). As species abundance in cities is known to be heavily influenced by human-subsidized biogeochemical flows (DeStefano and DeGraaf 2003), enhanced self-sufficiency may have ecosystem consequences both within the city and along urban rural gradients. Therefore, to better inform policy and practice, an integrated research approach that links urban material and energy flows to ecosystem consequences is needed.

### Intra-system heterogeneity

Spatial heterogeneity is an important concept in studying urban systems, and plays important roles in the functioning of ecological systems in general (Grimm et al. 2000; Luck and Wu 2002; Alberti 2005). So far studies on spatial heterogeneity are mostly focused on land use (Cadenasso et al. 2007), flora and fauna (Pickett et al. 2001), and the influence of income and other socioeconomic indicators (Kinzig et al. 2005; Pickett et al. 2011). In the urban metabolism literature, spatial heterogeneity is studied with the motivation to better inform the location of potential resources (see for example (Kapur and Graedel 2006)), or the relationship between spatial structure of cities on metabolism, with the motivation to identify planning implications but with mixed findings (Baker et al. 2001; Kennedy et al. 2009; Fissore et al. 2011; Heinonen and Junnila 2011; Liu et al. 2012). Increasing attention is being paid to social economic heterogeneity, i.e., how social differentiation and household behaviours can shape metabolic flows. Evidence shows that there is intra-system homogeneity in developed cities within the same region especially when embodied flows are taken into account (Minx et al. 2013). Much larger intra-city heterogeneity is observed in some developing cities (Lin et al. 2013), but little empirical evidence exists. Combining the two groups of literature together will enable a more comprehensive understanding of urban system heterogeneity, by adding more layers and opening up the potential to study interrelations among specific heterogeneities.

### Temporal and intercity variation

Cities are dynamic and evolving systems, and understanding the processes and mechanisms of changing systems and their environment and ecosystem consequences are one of the key tasks of urban research. Most flow budgets and intensity show an increasing trend over time (Warren-Rhodes and Koenig 2001; Kennedy et al. 2007; Baynes and Bai 2012), despite an increasing efficiency (Zhang and Yang 2007; Browne et al. 2009). The magnitude of the flows varies according to the development stages of cities. For example, up to 10–30-fold increase in construction material flows has been observed over 30 years in Beijing (Hu et al. 2010a, b). Income level determines the level of housing stocks across different cities in China, Norway, and the Netherlands (Hu et al. 2010a, b). There are large intercity disparities (Kennedy et al. 2009; Grubler et al. 2012), but larger disparity is in direct flows than embodied flows in developed cities with income and other socioeconomic variables as key determining factors (Minx et al. 2013). Intercity variation can be driven by functional differences, urban planning and management, and socioeconomic factors (Li et al. 2012).

### Regulating capacity

Understanding the biophysical and social mechanisms behind resource distribution is a common aspiration for urban ecosystem studies and urban metabolism studies (Batty 2008; Pickett et al. 2011; Chen and Chen 2012). Urban policy and governance practices can play significant role in shaping and regulating the metabolism (Heynen et al. 2006; Kaye et al. 2006; Bai 2007; Brunner 2007), and can drive improvements in flow efficiency (Zhang and Yang 2007). Some cities attempt to actively regulate urban metabolism, e.g., reducing total water consumption or pledging to reduce carbon emissions. On the one hand, it is important to note that such regulating capacity of cities are not without limit, due to the temporal, spatial, and institutional scale mismatches between urban management and the global extent of the flows (Bai et al. 2010). On the other hand, narrowly focused policy actions on reducing pollution alone may lead to the relocation of energy intensive and polluting industries outside of the city and into ecologically fragile areas (Bai et al. 2010). To avoid unintended negative consequences, a careful articulation of the purpose and impacts of the regulating capacity, and in doing so taking into account both ecosystem and energy/material flow concerns, is essential. Such self-regulating capacity through urban governance is a unique and important feature of urban ecosystems, which can be a powerful leverage to shift the system towards sustainability. More conceptual and empirical work is needed to better understand this feature of urban ecosystems.

## Concluding remarks

This study aims to seek a conceptual common ground between the urban metabolism studies and urban ecosystem studies. As demonstrated in this paper, while the conceptual starting points are very different, the two research communities share many common aspirations and foci, and are not exclusive. They can ask common questions and it is mutually beneficial and indeed possible to develop intellectual linkages. Important urban ecosystem insights, such as the eight key characteristics of urban ecosystems presented in this paper, that can be derived from urban metabolism studies, which in turn can be of significance for both urban ecology and industrial ecology communities. There are varying degrees of research coverage on these eight key aspects. Much less is known of the behaviour of these eight key aspects in relation to each other, and in relation to ecological processes in urban system, which can be important future research focus.

Integrating plural concepts, theories, and approaches will help inform theoretical development around cities as unique ecosystems. This is recognized by both urban metabolism and urban ecosystem communities, and there are some efforts to achieve broader integration including these two communities. For example, the social-ecological-infrastructure systems framework presented by Ramaswami et al. (2012) includes both urban ecology and urban metabolism in it, although the relative positioning of urban ecology within urban metabolism does not reflect the *ecology of cities* perspective, and Broto et al. (2012) identifies several cross-disciplinary synergies around the concept of urban metabolism. But such recognition and effort are not necessarily linked to concrete actions such as closely examining and cross-referencing advances from the other community. As shown in this paper, significant ecological insight can be, or has the potential to be, drawn from the rich and rapidly growing empirical findings of urban metabolism studies to understand the behaviour of cities as human-dominated, complex systems. Better integration will require some conscious efforts from both communities. To realize its full potential, urban metabolism studies need to be more conscious of the conceptual and theoretical development of urban systems studies. Meanwhile, it perhaps is time for urban ecosystem study to expand from human influenced ecological process to include the purely anthropogenic materials and energy flows as its key area of study. While similar argument was put forward previously (Grimm et al. 2000; Churkina 2008), the focus was still on the biogeochemical cycles of key nutrients such as C, N, P, and not on the study of the flows in the form of other materials including products and wastes. A stronger integration of the two communities is not a purpose in itself, but a starting point of exploring new conceptual, theoretical, and empirical understanding of urban systems beyond the two communities. For example, how the material and energy flow efficiency of urban ecosystem, and associated policy measures targeting, interact with other urban system attributes such as resilience?

A better intellectual linkage between urban energy and material flows and ecological processes has important policy implications. For example, while one of the most cited ecosystem services from a green park in cities is its carbon sequestration ability, an energy flow analysis in Montjuïc Park in Barcelona, Spain shows that the land area required to absorb the carbon emission from service sector activities to maintain the park would exceed 12 times the area of the park (Oliver-Solà et al. 2007). Such finding does not deny the many other benefits of an urban park, e.g., on biodiversity, employment related to the service activities, and human health and wellbeing, but calls for a more informed argument and conscious decision, where insights from urban metabolism analysis can contribute. Likewise, policies solely focusing on reducing urban energy and material flows and improving self-dependency may have unintended ecosystem impacts within and along the urban–rural gradient by altering current nutrient and energy subsidence structure. With unprecedented urbanization and associated landscape, economic, social, and cultural changes anticipated in developing world (Bai et al. 2014), there is an urgent and increasing demand for research to inform urban policy and management practice. A better understanding of the interactions between anthropogenic material and energy flows and ecosystem processes can help reduce unintended consequences of narrowly focused policy and management decisions.
